# Development and Assessment of a New Framework for Disease Surveillance, Prediction, and Risk Adjustment: The Diagnostic Items Classification System

**DOI:** 10.1001/jamahealthforum.2022.0276

**Published:** 2022-03-25

**Authors:** Randall P. Ellis, Heather E. Hsu, Jeffrey J. Siracuse, Allan J. Walkey, Karen E. Lasser, Brian C. Jacobson, Corinne Andriola, Alex Hoagland, Ying Liu, Chenlu Song, Tzu-Chun Kuo, Arlene S. Ash

**Affiliations:** 1 Boston University, Boston, Massachusetts; 2 Boston University School of Medicine, Boston, Massachusetts; 3 Massachusetts General Hospital and Harvard University, Boston; 4 Government Accountability Office, Washington, DC; 5 BMC HealthNet Plan, Boston, Massachusetts; 6 University of Massachusetts Medical School, Worcester

## Abstract

**Importance:**

Current disease risk-adjustment formulas in the US rely on diagnostic classification frameworks that predate the *International Classification of Diseases, Tenth Revision, Clinical Modification (ICD-10-CM)*.

**Objective:**

To develop an *ICD-10-CM*–based classification framework for predicting diverse health care payment, quality, and performance outcomes.

**Design, Setting, and Participants:**

Physician teams mapped all *ICD-10-CM* diagnoses into 3 types of diagnostic items (DXIs): main effect DXIs that specify diseases; modifiers, such as laterality, timing, and acuity; and scaled variables, such as body mass index, gestational age, and birth weight. Every diagnosis was mapped to at least 1 DXI. Stepwise and weighted least-squares estimation predicted cost and utilization outcomes, and their performance was compared with models built on (1) the Agency for Healthcare Research and Quality Clinical Classifications Software Refined (CCSR) categories, and (2) the Health and Human Services Hierarchical Condition Categories (HHS-HCC) used in the Affordable Care Act Marketplace. Each model’s performance was validated using *R*^2^, mean absolute error, the Cumming prediction measure, and comparisons of actual to predicted outcomes by spending percentiles and by diagnostic frequency. The IBM MarketScan Commercial Claims and Encounters Database, 2016 to 2018, was used, which included privately insured, full- or partial-year eligible enrollees aged 0 to 64 years in plans with medical, drug, and mental health/substance use coverage.

**Main Outcomes and Measures:**

Fourteen concurrent outcomes were predicted: overall and plan-paid health care spending (top-coded and not top-coded); enrollee out-of-pocket spending; hospital days and admissions; emergency department visits; and spending for 6 types of services. The primary outcome was annual health care spending top-coded at $250 000.

**Results:**

A total of 65 901 460 person-years were split into 90% estimation/10% validation samples (n = 6 604 259). In all, 3223 DXIs were created: 2435 main effects, 772 modifiers, and 16 scaled items. Stepwise regressions predicting annual health care spending (mean [SD], $5821 [$17 653]) selected 76% of the main effect DXIs with no evidence of overfitting. Validated *R*^2^ was 0.589 in the DXI model, 0.539 for CCSR, and 0.428 for HHS-HCC. Use of DXIs reduced underpayment for enrollees with rare (1-in-a-million) diagnoses by 83% relative to HHS-HCCs.

**Conclusions:**

In this diagnostic modeling study, the new DXI classification system showed improved predictions over existing diagnostic classification systems for all spending and utilization outcomes considered.

## Key Points

**Question:**

How can diagnostic information in the *International Classification of Diseases, Tenth Revision, Clinical Modification (ICD-10-CM)* be organized to improve the accuracy and usefulness of predictive models used for plan payment and disease surveillance?

**Findings:**

This diagnostic modeling study used insurance claims for 65 901 460 privately insured adults and children in the US from 2016 to 2018 to create new diagnostic items using *ICD-10-CM* codes that achieved a validated *R*^2^ almost 1.5 times that of Affordable Care Act Marketplace risk-adjustment model, with meaningful improvements for other outcomes.

**Meaning:**

Rich multidimensional diagnostic classification systems can improve predictive models for performance benchmarking and risk adjustment.

## Teaser

This diagnostic modeling study develops an *ICD-10-CM*–based classification framework for predicting diverse health care payment, quality, and performance outcomes.

## Introduction

Health systems use diagnostic codes for individual patient care as well as to validate insurance claims, calculate risk-adjusted health plan payments, establish case-mix indices, track disease prevalence, and evaluate clinician performance. In October 2015, the US expanded the number and precision of diagnoses available for coding patient conditions by more than 5-fold when it transitioned from the ninth to the tenth revision of the *International Classification of Diseases, Tenth Revision, Clinical Modification (ICD-10-CM)*.^1^ While the Agency for Healthcare Research and Quality (AHRQ) Clinical Classifications Software Refined (CCSR)^2^ incorporates certain features of the new *ICD-10-CM* codes, it largely still reflects its origin in the *International Classification of Diseases, Ninth Revision, Clinical Modification *structure and does not capture the full richness of the increased detail available in the *ICD-10-CM* system.

In this diagnostic modeling study, we developed novel diagnostic items (DXIs), a new classification system that leveraged the additional information in the *ICD-10-CM* system in 4 ways. First, many individual diagnoses were mapped into multiple DXIs, taking advantage of *ICD-10-CM*’s richer diagnosis-level information. Second, DXIs were ex ante designed to predict multiple outcomes, including spending, admissions, quality measures, and emergency department use. Third, DXIs were chosen to explain differences between realized outcomes and predicted values within subgroups defined by an existing base model—the AHRQ CCSR.^2^ Finally, DXIs were calibrated using very large sample sizes to enable robust estimation of the incremental influence of disease categories that are as rare as 1 in 100 000.

Several existing classification systems map diagnoses to categories. The World Health Organization has created and updates the international *ICD-10* coding system, which contains 21 chapters and finer subchapters that are comprehensive but not organized to predict costs or utilization.^3^ The Health and Human Services Hierarchical Condition Category (HHS-HCC) system^4^ was developed for the Medicare Advantage program, revised for Medicare Part D, and further expanded for plan payment in the Affordable Care Act Marketplace. Our effort builds on the comprehensive and up-to-date AHRQ CCSR system that managed care plans, insurers, researchers, and surveillance programs use for myriad applications related to payment, quality assessment, and epidemiology.^2^

Several commercial groupers are also available, although they do not fully document their methods in published research.^5,6^ These include the Johns Hopkins Adjusted Clinical Groups^7^ system that used 282 expanded diagnosis clusters for prediction^8^; the 3M Clinical Risk Groups system^9^; the DxCG classifications that substantially expand the detail available in HHS-HCCs^10^; and the Chronic Illness and Disability Payment System that is used by several state Medicaid programs.^11^ Although several articles have documented efforts to accommodate and extract value from the transition to *ICD-10-CM*,^12,13,14,15^ none of these systems has been fundamentally restructured.^16,17^ Our objective was to create a clinically detailed, transparent, well-documented, nonproprietary classification system suitable for predicting diverse outcomes using *ICD-10-CM* diagnostic information and share a core set of predictive models that can be used on other data sets and populations.

## Methods

### Study Sample

We used deidentified IBM/Watson Truven Commercial Claims and Encounters data spanning 2016 through 2018 in this diagnostic modeling study.^18^ The sample includes all enrollees aged 0 to 64 years who were enrolled for at least 1 month in noncapitated insurance plans with both pharmacy and medical coverage including treatment of substance use and mental health disorders. To detect and quantify overfitting, we reserved a randomly selected 10% sample (n = 6 604 259) of the available data (n = 65 901 460) for validation, leaving 90% (n = 59 297 201) for model development. Theoretical arguments suggest that the size of our validation sample is sufficient for providing stable findings.^19^

The Institutional Review Board of Boston University determined this study exempt from review because the secondary data used were deidentified (protocol 4973X). The database had no missing values and did not require follow-up. This study followed the Standards for Reporting of Diagnostic Accuracy (STARD) reporting guidelines for diagnostic studies.^20^

#### Data Filtering

We followed the filtering criteria used in the Marketplace HHS-HCC model, limiting diagnoses to those coded by acceptable health care professional types as defined by hospital inpatient, hospital outpatient, clinician specialty, and procedure codes.^4^ Previous work has revealed only small changes in rates of disease prevalence associated with HHS-HCC filtering.^21^ The eMethods in the Supplement contains additional details on data filtering, creation of DXIs, types of items created, and definition of diagnostic frequencies rates.

#### Creation of DXIs

We grouped all *ICD-10-CM* diagnoses as of October 2019 into new clusters that we call diagnostic items, or DXIs. The mappings included all 71 934 billable *ICD-10-CM* diagnosis codes and their 22 512 frequently nonbillable root stems. We included root codes to facilitate future applications of our mappings in countries not using the US “clinically modified” *ICD-10* code expansions. Owing to their pressing relevance, we also included the 2020 emergency use *ICD-10-CM* codes for COVID-19 and vaping-related disorders.

Assignment of DXIs took place between March 2019 and July 2021. The 5 physician coauthors (H.E.H., J.J.S., A.J.W., K.E.L., B.C.J.) assigned DXI categories, with assistance from clinical content experts when needed. To create DXI assignments, we consulted World Health Organization chapters and identified clusters of mutually exclusive diagnoses that (1) were clinically distinct, (2) had similar average concurrent and subsequent year spending, and (3) resulted in similar unexplained residuals when applied to a concurrent regression model predicting top-coded health care spending using the October 2018 beta version of the AHRQ CCSR system. The full set of figures used in the creation of the DXIs is available online at http://tinyurl.com/DXI-ICD10CM-Figures; eFigures 1 and 2 in the Supplement show the counts of *ICD-10-CM* diagnoses by number of DXIs and CCSR categories, respectively.

We created 3 types of DXIs. The primary or main effect DXIs, called DXI_1, focus on clinical dimensions in each diagnosis. Diagnoses were assigned up to 4 DXI_1s. In some cases, we created both broader and narrower DXI_1s that overlapped because we did not know a priori the level of detail preferred for prediction. We illustrate this approach below in our discussion of sepsis and hypertension in pregnancy DXI_1s.

The second group, DXI_2 modifiers, cut across DXI_1s. Some identify disease severity, such as “with complications,” “hemorrhage,” “secondary,” “bilateral,” and “with coma.” Others may be useful for disease monitoring, including flags for future research and epidemiological surveillance, such as sexually transmitted and vaccine-preventable infectious diseases. Certain diagnoses for external causes and factors influencing health status (whose codes begin with V-Z) were not assigned a DXI_1 and were instead only assigned DXI_2 modifiers.

Finally, DXI_3 scaled variables capture test results, disease severity, or clinically relevant distinctions not easily captured in binary DXI_1 categories. These include body mass index (BMI; calculated as weight in kilograms divided by height in meters squared), neonatal birth weight, neonatal gestational age, pregnancy trimester, low vision/blindness stages, coma scale measures, stroke scores, and duration of unconsciousness. As an example, the DXI_3 variable for BMI, calculated as weight in kilograms divided by height in meters squared, takes on values between 18.5 and 70, corresponding to ordered groups of BMI ranges. When comparing the DXI classification system to existing models, we included only main effects (DXI_1s) as predictors. This comparison cleanly demonstrates the value of the DXIs richer classification of diagnoses. Quantifying the additional value of using DXI_2 and DXI_3 items is left for future research.

The DXIs were developed by augmenting the May 2020 (version 2020.3) AHRQ CCSR classification system because it comprehensively mapped all *ICD-10-CM* codes and had more categories (540) than the HHS-HCC, which recognized only 14% of all diagnoses (9757 diagnosis codes) and used only 127 categories for prediction.^4^ Furthermore, the HHS-HCC sample frequencies and rationale for disease category inclusion or exclusion were not publicly available. The HHS-HCC model embedded clinical judgment about which diagnoses are appropriate to use for payment, which may not be the correct approach for other uses. Its fixed set of hierarchies and coarse set of diagnostic groups may do poorly in predicting other outcomes, such as quality measures used for performance assessment or benchmarking.^22^

### Outcomes

The DXIs are intended to be flexibly used for many purposes, including surveillance, understanding plan and clinician performance, and quality assessment. We focused model development on creating DXIs useful for measuring biased selection as well as for plan and health care professional payment, with our primary outcome being total annual spending for individual enrollees.^22^ During data cleaning, we recoded total spending by enrollee-year to $0 when it was negative, and to $3 million when it was larger. To limit the potentially large influence of outliers on means and coefficients on rare conditions, we further top-coded spending variables at $250 000 in our primary specification. Other spending outcomes included plan paid spending top-coded at $3 million and $250 000, and enrollee out-of-pocket spending top-coded at $500 000.

We annualized each outcome for all non-newborns so that the outcome is a rate per 12-month period and weighted observations in regressions based on the fraction of the year each enrollee was observed.^4,23,24^ We did not use this procedure for newborns, given their high levels of spending at birth; rather, we set their regression weights to 1. We converted all spending into 2018 dollars using the consumer price index. We also estimated models to predict utilization outcomes: counts of inpatient admissions, inpatient days, emergency department visits,^25^ and plan payments for 6 service types (inpatient and outpatient facility pharmacy prescriptions, outpatient retail prescriptions, imaging, laboratory, and preventive care visits). The definitions of these utilization outcomes are included in eTable 1 in the Supplement.

We incorporated DXI_1s into a concurrent payment prediction model, in which diagnoses and other clinical information within a year were used to predict outcomes for that same year. Concurrent models are currently implemented in the Affordable Care Act (ACA) Marketplace and many Medicaid programs in the US and are more robust to data limitations. We do not present here any results based on a prospective model, as is used in the Medicare risk-adjustment model, because that would require different data configuration, sample selection, and HCCs. We calculated all performance measures in the 10% validation sample.

### Statistical Analysis

We estimated unconstrained weighted least-squares and stepwise regression models (with an inclusion criterion of *P* < .0001) that predicted concurrent outcomes (1) using only age and sex variables, (2) HCC variables, (3) CCSR variables,^2^ and (4) our DXI framework. The significance of individual coefficients and their confidence intervals were calculated using the Bonferroni correction for the large number of parameters considered in each model specification. We compared model performance using validation sample measures of *R*^2^. For utilization measures, we also calculated the mean absolute errors and the Cumming prediction measures, which we modified from their conventional specification to reflect the sample weighting used to correct for partial-year enrollees. We also examined how well models distinguish between enrollees with common vs rarely occurring diagnoses in the validation sample to quantify the potential profitability of successfully avoiding coverage of people with rare conditions. All statistical analysis was performed using SAS, version 9.4 (64 bit) (SAS Institute).

## Results

We created 3223 DXIs: 2435 DXI_1 main effects, 772 DXI_2 modifiers, and 16 DXI_3 scaled variables. Full details of the mappings of *ICD-10-CM* codes into DXIs are available online at http://tinyurl.com/DXI-Mappings.

The 90% development sample included 59 297 201 enrollee-years. Mean (SD) total health care and plan paid spending were $6124 ($25 109) and $5281 ($24 585), respectively, with no meaningful differences between the development and estimation samples (eTable 2 in the Supplement). Mean (SD) total health care spending top-coded at $250 000 (the primary outcome) within the development sample was $5821 ($17 653); top-coding lowered mean total health care spending by 4.9%.

### DXI Case Studies

**Figure 1.**
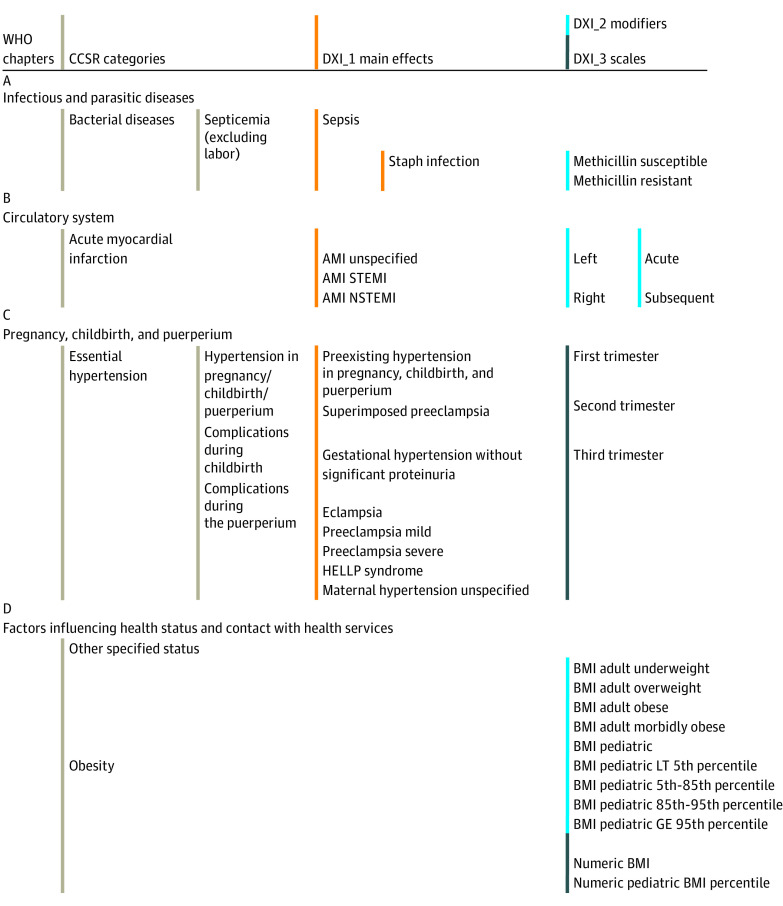
Examples Illustrating the DXI Classification Structure AMI indicates acute myocardial infarction; BMI, body mass index (calculated as weight in kilograms divided by height in meters squared); CCSR, Clinical Classifications Software Refined v2019.1 (beta version); DXI, diagnostic item; GE, greater than or equal to; HELLP, hemolysis, elevated liver enzyme and low platelet; LT, less than; NSTEMI, non–ST-segment elevation myocardial infarction; STEMI, ST-segment elevation myocardial infarction; WHO, World Health Organization.

Figure 1 provides a schematic framework for mapping individual *ICD-10-CM* codes to DXIs, illustrating the precision in classification enabled by the *ICD-10-CM* system. For example, Figure 1A includes example DXI_1s that distinguish between staphylococcus infections that are methicillin susceptible and methicillin resistant, which proves to be meaningful in predicting spending. A total of 3136 cases of “Sepsis due to Methicillin susceptible Staphylococcus aureus” were underpredicted by $15 350 by the CCSR model (http://tinyurl.com/DXI-ICD10CM-Figures); using finer DXI categories for sepsis ameliorated this underprediction. Similarly, large variations were identified in the costs associated with patients with acute myocardial infarction (http://tinyurl.com/DXI-ICD10CM-Figures), which motivated the separation of ST-segment elevation myocardial infarction from non–ST-segment elevation myocardial infarction and unspecified acute myocardial infarction illustrated in Figure 1. Further differences are apparent between ST-segment elevation myocardial infarction with left vs right coronary artery involvement motivating the distinctions in DXI_2 for laterality.

Although not presented in full here, the DXI classification system created DXI_2 and DXI_3 categories to incorporate additional information and capture variation within a specific clinical condition. For example, Figure 1C illustrates how DXI modifiers can distinguish among common pregnancy-related complications, as well as allow for variation across pregnancy trimesters. Finally, Figure 1D illustrates how a continuous modifier—BMI—can potentially explain spending and clinical outcomes beyond the CCSR’s current diagnostic categories that simply identify obesity.

### Linear Regression Models for Selected Outcomes

Table 1 presents validation sample *R*^2^ results from 5 spending outcomes. The age-sex models included 29 age-sex demographic dummy variables and achieved *R*^2^s of 0.013 to 0.040, consistent with prior research.^23,24^ The HCC model performed substantially better than the age-sex model, but the CCSR model improved the *R*^2^ above the HCC model by 0.08 or more for each spending outcome. The DXI model, which added 2435 main effect DXIs to the CCSR categories, further increased the *R*^2^ by 0.05 or more for every outcome except out-of-pocket spending, where it added only 0.019. These measures vary little across the development and validation samples, owing to large overall and within-DXI sample sizes, resulting in minimal overfitting (eTable 3 in the Supplement). Finally, the bottom row of Table 1 shows that stepwise regression reduced the number of variables by 23% to 29%, with no detectable change in predictive power.

**Table 1.** Validated *R*^2^s for Predicting 5 Spending Outcomes^a^

Outcome	OLS				Stepwise OLS^b^
	Age-sex only	HCC	CCSR	DXI	DXI
Spending measures, $					
Total health care	0.015	0.349	0.438	0.510	0.510
Total health care top-coded at 250 000	0.026	0.428	0.539	0.589	0.589
Plan paid	0.013	0.341	0.426	0.499	0.499
Plan paid top-coded at 250 000	0.023	0.421	0.527	0.578	0.578
Out of pocket	0.040	0.186	0.310	0.329	0.329
No. of explanatory variables	29	166	567	2929	2079-2245^c^

Abbreviations: CCSR, Clinical Classifications Software Refined model; DXI, diagnostic items model; HCC, Hierarchical Condition Category model; OLS, ordinary least squares.

^a^ All models included age and sex as adjusters. All models were estimated using the development sample with n = 59 297 201. These validation sample measures used n = 6 604 259.

^b^ The stepwise regression used in the final column used *P* < .0001 for variable inclusion.

^c^ The number of variables selected by stepwise OLS varied with the spending measure over this range.

Full sets of regression results for top-coded and not top-coded total spending are available at http://tinyurl.com/DXI-StepwiseOLS. Of note, many of the regression coefficients were negative, which is not surprising given the substantial collinearity among non–mutually exclusive DXI and CCSR terms. These negative coefficients on individual terms are generally offset by positive coefficients on related measures. Negative coefficients are not as concerning as negative predictions, which reflect the net effect of all variables that each enrollee is coded with. Using the validation sample, 4.47% were assigned negative spending for top-coded spending, and 5.46% for not top-coded spending. If these negative amounts were not allowed, it would change the means for the total spending models by less than 0.5%. These findings are discussed further in eMethods in the Supplement.

Table 2 presents fit statistics for 9 clinical outcomes. The DXI models improved on the *R*^2^ by more than 10% over the CCSR model in every case, with sizeable improvements also observed for the mean absolute error and the Cumming prediction measure across almost every outcome. The Cumming prediction measure was negative for the CCSR model for inpatient spending on prescription drugs in the validation sample, although less negative (ie, better) for the DXI model. Mean predictions and predictive ratios for the DXI model compared with the HCC and CCSR models across percentiles of actual spending are presented in eTable 4 in the Supplement, with meaningful improvement in the upper percentiles where concerns about underprediction are the most concerning.

**Table 2.** Goodness-of-Fit Measures for CCSR and DXI Models on 9 Utilization Measures^a^

Outcome variables	CCSR OLS			DXI OLS^b^		
	*R* ^2^	Mean absolute error	Cumming prediction measure	*R* ^2^	Mean absolute error	Cumming prediction measure
Count variables						
IP admissions	0.507	0.063	0.384	0.565	0.057	0.442
IP days	0.379	0.370	0.146	0.479	0.310	0.284
ED visits	0.329	0.273	0.306	0.383	0.260	0.342
Spending by type of service, $						
IP facility pharmacy	0.134	212	−0.339	0.187	191	−0.204
OP facility pharmacy	0.170	569	0.056	0.208	547	0.093
Retail pharmacy	0.205	1480	0.258	0.238	1431	0.283
Laboratory	0.234	582	0.179	0.268	564	0.203
Imaging	0.308	1587	0.323	0.380	1391	0.407
Preventive care visits	0.573	39	0.581	0.637	33	0.647
No. of explanatory variables	567	567	567	2929	2929	2929

Abbreviations: CCSR, Clinical Classifications Software Refined model; DXI, diagnostic items model including CCSR variables; ED, emergency department; IP, inpatient; OLS, ordinary least squares; OP, outpatient.

^a^ All models also included 29 age-sex dummy variables. Measures are all concurrent measures, annualized and weighted by the fraction of the year eligible, using the validation sample (n = 6 604 259).

^b^ The DXI models included both main effects DXI_1s and CCSR variables.

Table 3 compares the DXI model to the HCC and CCSR models in numbers of regressors, both overall and those which are statistically significant (*P* < .001). For example, across the eye, ear, and skin disease chapters—comprising more than 4000 diagnoses in total—the FY2018 HCC model recognized only 1 disease category, and the CCSR recognizes 25 categories, while our DXI system uses 378 DXIs. Other chapters with large increases in the numbers of significant coefficients are infectious and parasitic diseases, blood disorders, diseases of the nervous system, and musculoskeletal conditions.

**Table 3.** Numbers of Categories in the HCC, CCSR, and DXI Classification Systems

WHO chapter	*ICD* code range	Chapter abbreviation	Chapter label	Valid *ICD-10-CM* code	HHS-HCC^a^	CCSR	DXI^b^	Statistically significant DXI^c^
1	A00-B99	INF	Certain infectious and parasitic diseases	1058	5	12	114	66
2	C00-D49	NEO	Neoplasms	1661	6	74	206	133
3	D50-D89	BLD	Diseases of the blood and blood-forming organs and certain disorders involving the immune mechanism	247	9	10	47	42
4	E00-E89	END	Endocrine, nutritional, and metabolic diseases	908	10	17	83	66
5	F01-F99	MBD	Mental, behavioral, and neurodevelopmental disorders	747	9	32	150	123
6	G00-G99	NVS	Diseases of the nervous system	622	13	22	116	98
7	H00-H59	EYE	Diseases of the eye and adnexa	2606	0	12	240	121
8	H60-H95	EAR	Diseases of the ear and mastoid process	656	0	6	38	30
9	I00-I99	CIR	Diseases of the circulatory system	1350	14	39	88	77
10	J00-J99	RSP	Diseases of the respiratory system	341	4	17	65	56
11	K00-K95	DIG	Diseases of the digestive system	799	9	25	102	82
12	L00-L99	SKN	Diseases of the skin and subcutaneous tissue	846	1	7	100	56
13	M00-M99	MSK	Diseases of the musculoskeletal system and connective tissue	6487	6	38	206	179
14	N00-N99	GEN	Diseases of the genitourinary system	669	3	26	104	88
15	O00-O9A	PRG	Pregnancy, childbirth, and the puerperium	2267	14	30	153	84
16	P00-P96	PNL	Certain conditions originating in the perinatal period	443	NA^d^	15	51	38
17	Q00-Q99	MAL	Congenital malformations, deformations, and chromosomal abnormalities	817	4	10	34	30
18	R00-R99	SYM	Symptoms, signs, and abnormal clinical and laboratory findings, not elsewhere classified	720	2	17	172	128
19	S00-T88	INJ	Injury, poisoning, and certain other consequences of external causes	40 570	7	76	173	121
	U00-U99	SPL	Emergency code additions	2	0	0	2	
20	V00-Y99	EXT	External causes of morbidity	6865	0	30	28	11
21	Z00-Z99	FAC	Factors influencing health status and contact with health services	1253	11	25	163	131
			Totals	71 934	127	540	2435	1760

Abbreviations: CCSR, Clinical Classifications Software Refined model; DXI, diagnostic items model including CCSR variables; HHS-HCC, Health and Human Services Hierarchical Condition Categories model; *ICD*, *International Classification of Diseases*; *ICD-10-CM, International Classification of Diseases, Tenth Revision, Clinical Modification*; NA, not applicable; OLS, ordinary least squares; WHO, World Health Organization.

^a^ The HHS-HCC model coefficient counts were from the adult model. Each of the 127 HHS-HCCs included in the HHS risk-adjustment model were assigned to *ICD-10-CM* chapters based on their corresponding diagnosis codes. Each HCC was assigned to the *ICD-10-CM* chapter containing a plurality of its diagnosis codes.

^b^ The DXI counts excluded CCSR variables.

^c^ Statistically significant coefficient counts include all DXI categories whose coefficient (in a model predicting total health care spending top-coded at $250 000) met the Bonferroni-corrected threshold of *P* < .0001.

^d^ Neonatal codes distinguished in the HHS-HCC infant spending model are not included here.

### Improved Performance for Rare Diagnoses

**Figure 2.**
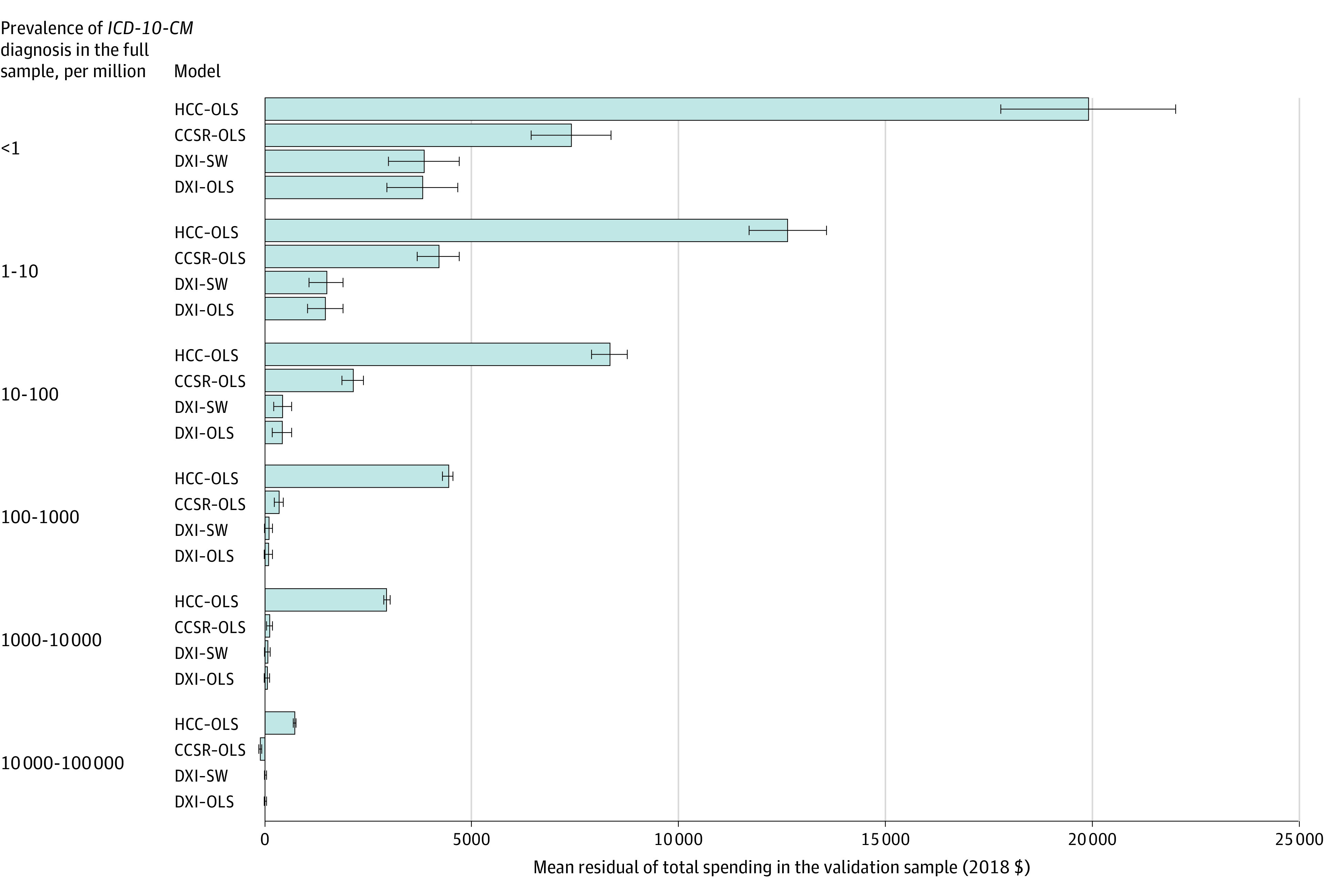
Mean Residuals of Total Spending for 4 Models by Diagnostic Frequency For the HCC, CCSR, and DXI models, we calculated the residuals from the total spending model at the enrollee-year level and then assigned these residuals to every unique *International Classification of Diseases, Tenth Revision, Clinical Modification (ICD-10-CM)* diagnosis each enrollee had in a year. We then calculated enrollee-weighted mean residuals in the validation sample using the binned frequencies of diagnoses in the full sample, with frequency intervals determined by powers of 10 per million. Plot whiskers correspond to 95% CIs, corrected for clustering at the patient level. CCSR indicates Clinical Classifications Software Refined model; DXI, diagnostic items model; HCC, Hierarchical Condition Category model; OLS, ordinary least squares; SW, stepwise.

Figure 2 compares average residuals for predicting total health care spending in the validation sample (n = 6.6 million) for HCC, CCSR, and DXI diagnosis-based risk-adjustment models by their diagnostic frequency in the full sample (n = 65.9 million) (eFigure 3 in the Supplement presents a similar figure for top-coded total spending). Although all systems show only modest errors for diagnoses appearing in at least 10 000 cases per million (1%) enrollee-years, mean residuals for rare diagnoses are often large. The DXI system residuals averaged 83% lower than HCC residuals for diagnoses occurring less than 1 time per million enrollee-years in the full sample, and even larger percentage improvements for diagnoses appearing once per 1000 to once per 100 000 enrollee-years.

## Discussion

In this diagnostic modeling study using claims data from privately insured enrollees, we created and validated a clinician-informed and data-driven diagnosis classification system that integrated the enhanced precision of the updated *ICD-10-CM* coding system. Our results demonstrate that a detailed diagnosis classification system can improve the predictive power of models for a wide range of outcomes used for setting health plan payments, performance assessment, risk adjustment, and benchmarking.

Our findings highlight that it is possible to substantially improve on the existing HHS-HCC and AHRQ CCSR models for health plan payment using a concurrent framework. For not top-coded plan spending, the AHRQ CCSR model improved predictive power over the HHS-HCC model by 26%, while the DXI model achieved a 46% improvement. These improvements are particularly salient when paying or benchmarking performance for patients with rare conditions.

Our findings are consistent with work exploring increasing model complexity in risk adjustment. For example, researchers in the Netherlands found nontrivial improvement using models allowing the mapping of individuals to multiple diagnosis-based cost groups, which outweighed the computational burden and overfitting risk of increased model complexity.^26^ Our detailed DXI main effect models added richness without meaningful overfitting. The improved predictions presented here are without using the additional information in the DXI_2 modifiers and DXI_3 scale variables.

Some have argued that building models on broad categories or narrow subsets of all diseases is adequate to ensure accurate predictions and fair payments.^27^ Our study showed that finer categories, such as the DXIs, improved model performance overall and are needed to improve predictions for enrollees with rare conditions. The DXIs reduced average errors by 80% to 90% relative to the HHS-HCC model for enrollees with rare (1-in-1000 to 1-in-1 000 000) diagnoses, as shown in Figure 2. Modeling with DXI categories thus fixes a concerning selection problem that remains even when the global fit of payments to expected costs is improved by other means, such as constrained regression, reinsurance, mixed payment, and outlier adjustments that have recently been proposed.^28^

### Limitations

Our results have several limitations. First, we limited our evaluations to examining the predictive power of concurrent models and have not explored the value of the DXI system in prospective modeling, as is used in Medicare’s risk-adjustment formulas. Second, these models created but did not evaluate the usefulness of DXI_2 modifiers or DXI_3 scaled variables, including information such as bilaterality, acuity, and timing. Third, we did not examine how to select which DXIs to include or exclude from a payment model, which previous research suggests can be done to improve incentives with little loss in predictive power.^24^ Fourth, the development data included only enrollees with private, employer-sponsored insurance; spending, coding, and treatment patterns may not be generalized to other populations. Fifth, we relied exclusively on linear regression models as is commonly done in contemporary risk adjustment. We did not explore other approaches, such as machine learning algorithms, constrained regressions, outlier constrained regression, or incorporating information about the appropriateness of including certain diagnostic information. Finally, we did not explore incorporating prescription drug diagnostic information, which is currently used in the ACA Marketplace risk-adjustment project. Prescription drug information can readily be added to the new system, as has been done for Medicare Advantage, the ACA Marketplace, and in other countries. Nonetheless, this study’s straightforward modeling provides a clear and unbiased assessment of the gains in power that can be achieved simply by using the new system’s highly detailed classification of diagnostic codes.

## Conclusions

This diagnostic modeling study describes and tests a new classification system that maps *ICD-10-CM* codes into a rich set of diagnostic items (referred to as DXIs), far more fully exploiting *ICD-10-CM*’s expanded diagnostic detail than widely used existing models. The DXI system predicts key spending and utilization outcomes more accurately than the existing models, potentially enabling improved plan payment, health services research, cost-effectiveness studies, quality reporting, and disease surveillance.

## Notes

Supplement. **eFigure 1.** Counts of ICD-10-CM Diagnoses by Number of DXIs **eFigure 2.** Counts of ICD-10-CM Diagnoses by Number of CCSR **eFigure 3.** Mean Residuals of Top-Coded Total Spending for Four Models, by Diagnostic Frequency **eTable 1.** Definitions of Utilization Measures **eTable 2.** Summary Statistics from the Development and Validation Samples **eTable 3.** R^2^ Using Development Sample for Five Spending Outcomes **eTable 4.** Mean Predicted Values and Predictive Ratios by Percentiles of Total Spending for 5 Models **eMethods.** DXI Design Features
