# Supplementary material for: Development and Assessment of a New Framework for Disease Surveillance, Prediction, and Risk Adjustment: The Diagnostic Items Classification System
Source: JAMA Health Forum. 2022 Mar 25;3(3):e220276. doi: 10.1001/jamahealthforum.2022.0276 (PMC8956982; doi:10.1001/jamahealthforum.2022.0276)
Supplement: Supplement. — eFigure 1. Counts of ICD-10-CM Diagnoses by Number of DXIs eFigure 2. Counts of ICD-10-CM Diagnoses by Number of CCSR eFigure 3. Mean Residuals of Top-Coded Total Spending for Four Models, by Diagnostic Frequency eTable 1. Definitions of Utilization Measures eTable 2. Summary Statistics from the Development and Validation Samples eTable 3. R2 Using Development Sample for Five Spending Outcomes eTable 4. Mean Predicted Values and Predictive Ratios by Percentiles of Total Spending for 5 Models eMethods. DXI Design Features [file jamahealthforum-e220276-s001.pdf]

Supplemental Online Content

Ellis RP, Hsu HE, Siracuse JJ, et al. Development and assessment of a new framework for disease surveillance, prediction, and risk adjustment: the diagnostic items classification system. *JAMA Health Forum*. 2022;3(3):e220276. doi:10.1001/jamahealthforum.2022.0276

eFigure 1. Counts of ICD-10-CM Diagnoses by Number of DXIs

eFigure 2. Counts of ICD-10-CM Diagnoses by Number of CCSR

eFigure 3. Mean Residuals of Top-Coded Total Spending for Four Models, by Diagnostic Frequency

eTable 1. Definitions of Utilization Measures

eTable 2. Summary Statistics from the Development and Validation Samples

eTable 3. R^2 Using Development Sample for Five Spending Outcomes

eTable 4. Mean Predicted Values and Predictive Ratios by Percentiles of Total Spending for 5 Models

eMethods. DXI Design Features

This supplemental material has been provided by the authors to give readers additional information about their work.

© 2022 Ellis RP et al. *JAMA Health Forum.*

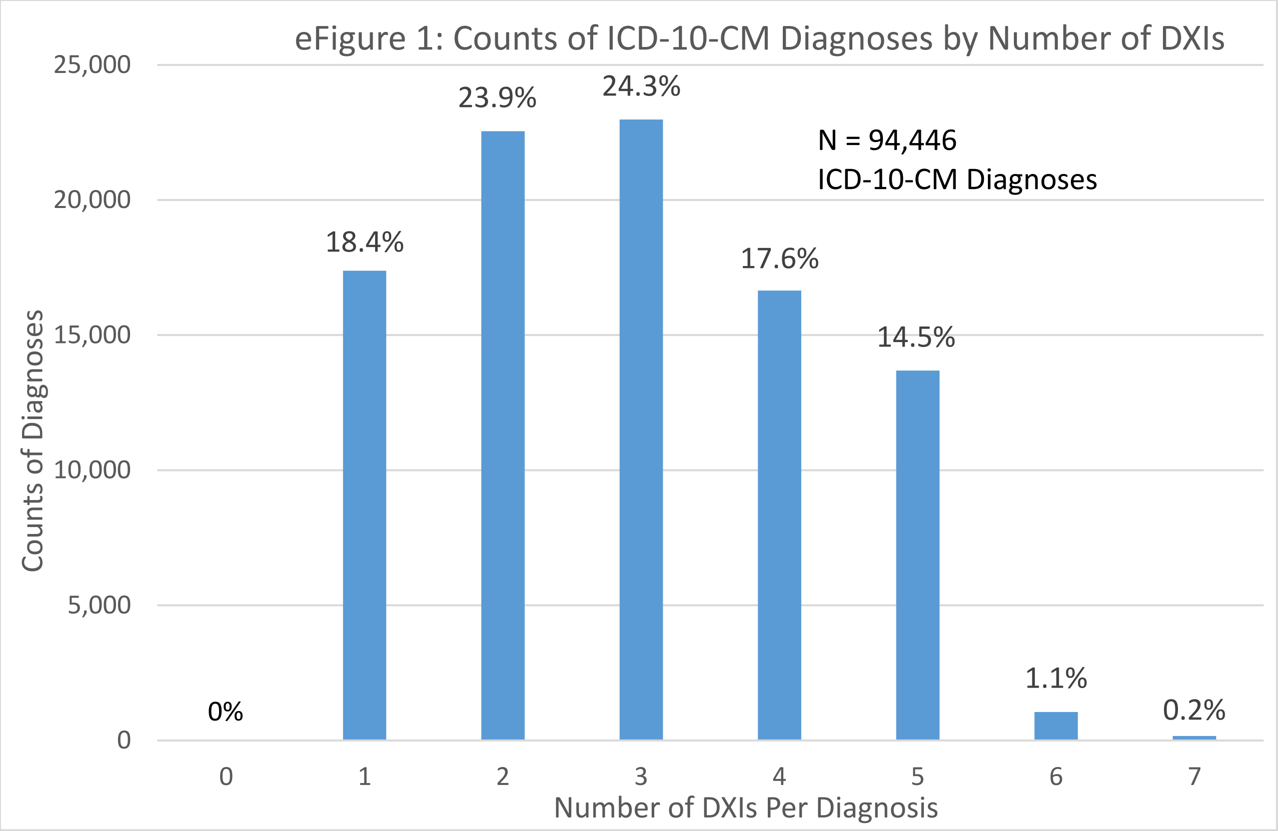

© 2022 Ellis RP et al. *JAMA Health Forum.*

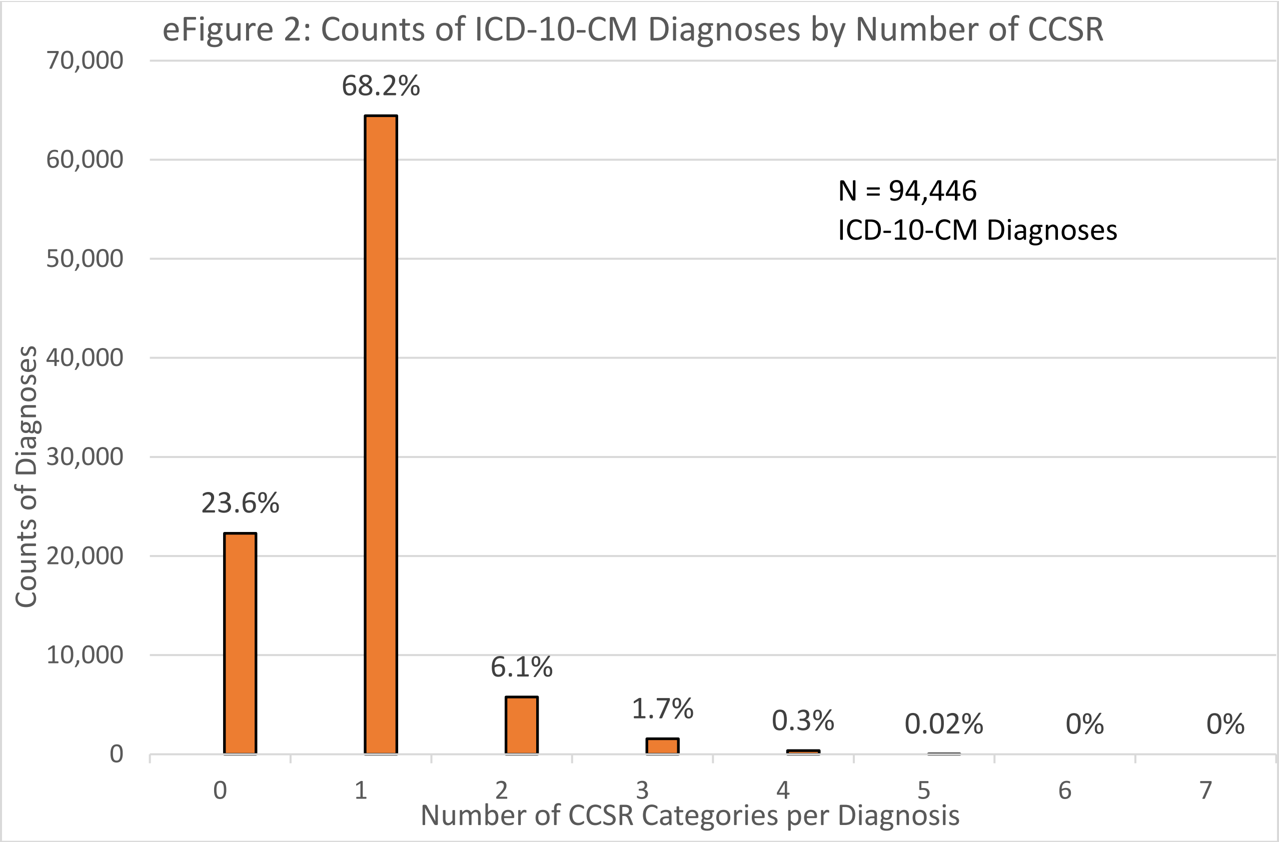

© 2022 Ellis RP et al. *JAMA Health Forum.*

eFigure 3. Mean Residuals of Top-Coded Total Spending for Four Models, by Diagnostic Frequency

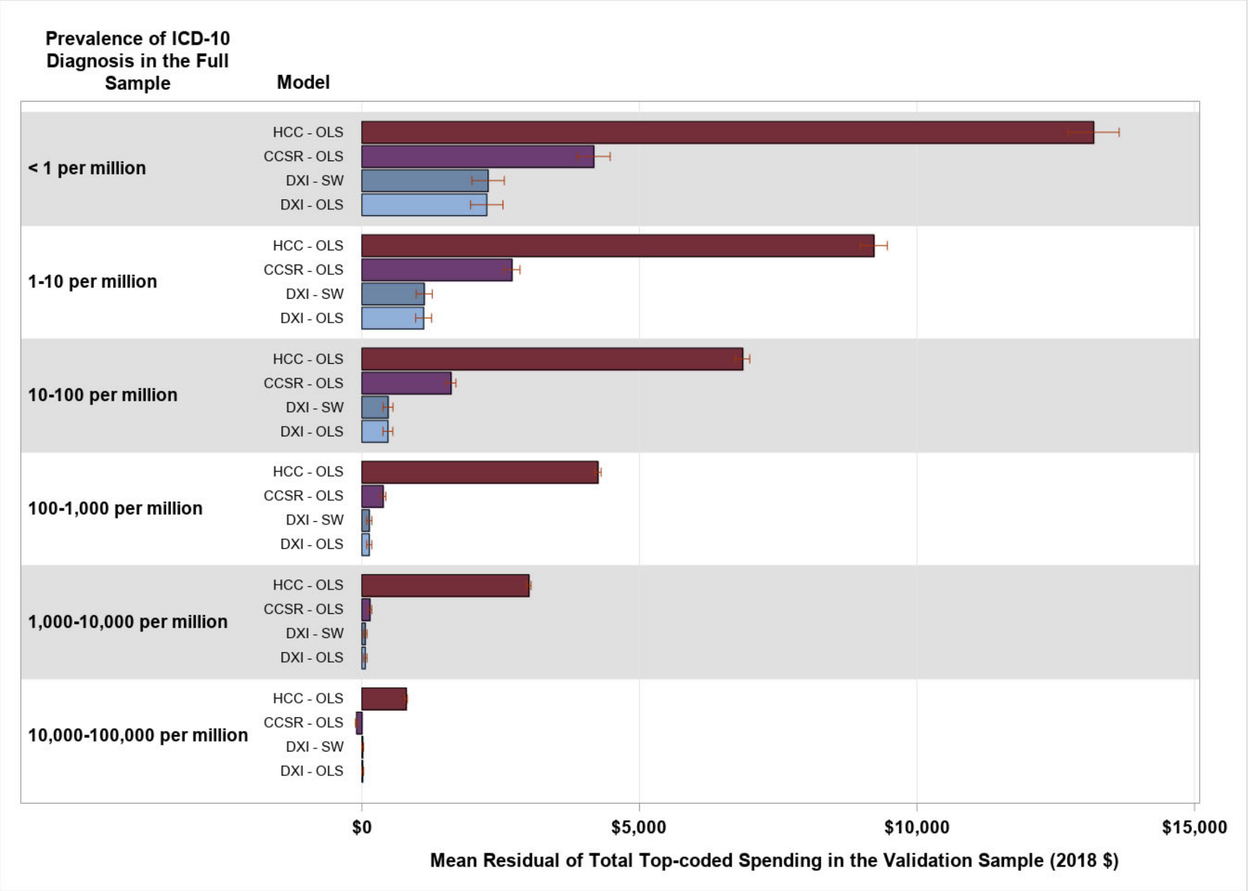

Notes: HCC is the Hierarchical Condition Category model, CCSR is the Clinical Classifications Software Refined model, DXI is the Diagnostic Items model, OLS is ordinary least squares, and SW is stepwise. For the HCC, CCSR, and DXI models, we calculated the residuals from the top-coded total spending model at the enrollee-year level and then assigned these residuals to every unique ICD-10-CM diagnosis each enrollee had in a year. We then calculated enrollee-weighted mean residuals in the validation sample using the binned frequencies of diagnoses in the full sample, with frequency intervals determined by powers of ten per million. Plot whiskers correspond to 95% confidence intervals, corrected for clustering at the patient level.

© 2022 Ellis RP et al. *JAMA Health Forum.*

eTable 1. Definitions of Utilization Measures

Variable	Definition
Inpatient admissions	The count of inpatient admissions by enrollee-year was defined as the count of distinct values of the CASEID variable from the Inpatient Admissions Tables I. The year of an inpatient admission was based on the date of admission. Watson Health used a proprietary admission construction methodology to group claims and encounters into inpatient admissions, which were uniquely identified in the Inpatient Admissions Tables I by the variable CASEID. [25, 26]
Length of stay	The length of stay (LOS) by enrollee-year-admission was defined as the difference between the discharge date and the admission date of an admission record in the Inpatient Admissions Tables I. The year was determined by the date of admission. Admissions with same-day discharges were assigned a LOS of one. Days of an inpatient stay which extended past the year of the admission date were excluded from the LOS. IBM Watson Health used a proprietary admission construction methodology to group claims and encounters into inpatient admissions.[25, 26]<br><br>There were 2 enrollee-years in the development sample that had a sum of LOS that exceeded 365 days (or 366 in 2016, a leap year). This was the result of admissions with overlapping dates.<br><br>In addition, there were enrollees with inpatient stays that included days that fell outside of the period of enrollment from the Enrollment Tables A. Payers may have provided coverage for inpatient stays which began during a period of enrollment and continued after the period of enrollment ended. Because the sum of LOS at the enrollee-year level were annualized using the months of enrollment from the Enrollment Tables A, this resulted in additional enrollee-years in the development sample that had annualized counts of LOS that exceeded 365 days (or 366 in 2016).<br><br>In total, there were 89 enrollee-years that had an annualized sum of LOS that exceeded 365 days (or 366 in 2016). To avoid this, we top-coded the annualized sum of LOS by enrollee-year at 366 in all years.

© 2022 Ellis RP et al. *JAMA Health Forum.*

Variable	Definition
Emergency Department Visits	Emergency department (ED) claims were identified using the last two characters of the service sub-category code in the SVCSCAT variable from the Outpatient and Inpatient Services Tables O and S, which corresponded to the service type. SVCSCAT values that have the last 2 characters "20" corresponded to ED visits.<br><br>There may have been multiple professional and facility claims associated with a single ED visit. ED claims with adjacent service dates for the same enrollee may have corresponded to a single overnight ED visit or distinct ED visits. To group ED claims into ED visits, we followed the Yale Operational Definition for ED Visitation presented in Venkatesh et al. (2017).[27] In this method, a professional ED claim is treated as a unique ED visit. ED facility claims which occur within ± 1 day of a professional ED claim are grouped with the professional ED claim. All other ED facility claims are treated as distinct ED visits.<br><br>Because multiple facility and professional claims may be associated with a single ED visit in the data, we grouped all ED claims by enrollee, date of service, and professional or facility category into day episodes before we applied this method. That is, a professional ED day episode was treated as a unique ED visit. ED facility day episodes which occurred within ± 1 day of a professional ED day episode were grouped with the professional ED day visit. All other ED facility day episodes were treated as distinct ED visits. Claims were classified as professional or facility using the variable FACPROF.<br><br>There were a small number of enrollees with a high number of ED visits with dates of service that fell outside of their period of enrollment from the Enrollment Tables A. Because the count of ED visits at the enrollee-year level were annualized using the months of enrollment from the Enrollment Tables A, this resulted in 2 enrollee-years in the development sample where the annualized count of ED visits exceeded 365 days (or 366 in 2016). To avoid this, we top-coded the annualized count of ED visits at 366 in all years.
Inpatient Facility Pharmacy Spending	IP facility and specialty drug claims were identified using the last two characters of the service sub-category code in the SVCSCAT variable from the Inpatient Services Tables S. The last two digits of SVCSCAT corresponded to the service type — "34" indicated facility pharmacy services and "36" indicated specialty drug services.

© 2022 Ellis RP et al. *JAMA Health Forum.*

Variable	Definition
Outpatient Facility Pharmacy Spending	OP facility and specialty drug claims were identified using the last two characters of the service sub-category code in the SVCSCAT variable from the Outpatient Services Tables O. The last two digits of a SVCSCAT value corresponded to the service type — "34" indicated facility pharmacy services and "36" indicated specialty drug services.
Outpatient Retail Pharmacy Spending	All records from the Outpatient Pharmaceutical Claims Tables D corresponded to retail pharmacy and mail-order drug claims.
Laboratory Spending	Laboratory claims were identified using the last two characters of the service sub-category code in the SVCSCAT variable from the Outpatient and Inpatient Services Tables O and S. The last two digits of a SVCSCAT value corresponded to the service type — laboratory services had "5" as the penultimate character. Laboratory services included chemistry tests, hematology, immunology, microbiology, pathology, urinalysis tests, and other laboratory services.
Imaging Spending	Imaging claims were identified using the last two characters of the service sub-category code in the SVCSCAT variable from the Outpatient and Inpatient Services Tables O and S. The last two digits of a SVCSCAT value corresponded to the service type — imaging services had "6" as the penultimate character. Imaging services included CT scans, mammograms, MRIs, nuclear medicine, PET scans, therapeutic radiology, ultrasounds, X-Rays, and other radiology services.
Preventive Care Visits Spending	Preventive care visits claims were identified using the last two characters of the service sub-category code in the SVCSCAT variable from the Outpatient and Inpatient Services Tables O and S. The last two digits of a SVCSCAT value corresponded to the service type — "24" indicated preventative care visit services.

© 2022 Ellis RP et al. *JAMA Health Forum.*

eTable 2. Summary Statistics from Development and Validation Samples

	Development Sample				Validation Sample		
	(N = 59,297,201)				(N = 6,604,259)		
Study variables	Mean		Std Dev		Mean		Std Dev
Demographic:							
Age	33.70		17.29		33.69		17.29
Female	51.48%				51.49%		
Months eligible	11.37		1.74		11.37		1.74
Spending measures:							
Total healthcare	$6,124		$25,109		$6,103		$24,782
Total healthcare top-coded at $250,000	$5,821		$17,653		$5,807		$17,649
Plan paid	$5,281		$24,585		$5,261		$24,256
Plan paid top-coded at $250,000	$4,985		$17,074		$4,972		$17,070
Out-of-pocket (OOP)	$844		$1,471		$843		$1,479
Count variables							
Emergency department (ED) visits	0.23		0.77		0.23		0.78
Inpatient days	0.23		2.40		0.23		2.37
Inpatient admissions	0.05		0.30		0.05		0.30
Spending by type of service:							
Inpatient facility pharmacy	$76		$2,239		$74		$2,035
Outpatient facility pharmacy	$295		$6,358		$296		$6,413
Outpatient retail pharmacy	$1,271		$7,393		$1,268		$7,357
Imaging	$357		$6,401		$356		$6,371
Laboratory	$1,656		$5,926		$1,653		$5,942
Preventive care visits	$76		$110		$76		$110

Notes: All outcomes are annualized and then weighted by the fraction of the year eligible for all enrollee-years except newborns. We recoded spending for the 0.008% of enrollee-years with negative total spending to zero, and the spending for the 0.016% with spending over three million dollars to three million dollars. Together these two adjustments lowered mean total spending and paid amounts by 0.051% and 0.059%, respectively. We recoded the spending by type of service for enrollee-years with spending over one million dollars for a given type of service to one million dollars.

© 2022 Ellis RP et al. *JAMA Health Forum.*

eTable 3. R^2 Using Development Sample for Five Spending Outcomes

	OLS					Stepwise OLS
	Age-sex only	HCC	CCSR	DXI		DXI
Spending measures:<br>Total healthcare	0.014	0.332	0.432	0.504		0.504
Total healthcare top-coded at $250,000	0.026	0.428	0.540	0.591		0.591
Plan paid	0.012	0.324	0.420	0.494		0.493
Plan paid top-coded at $250,000	0.023	0.422	0.527	0.580		0.580
Out-of-pocket (OOP)	0.040	0.182	0.313	0.332		0.332
Number of explanatory variables	29	166	567	2,929		2,079 to 2,245

Notes: All models included age and sex as adjusters. The stepwise regression used in the final column used P<.0001. Models were estimated using the development sample with N=59,297,201. DXI is the Diagnostic Items model, HCC is the Hierarchical Condition Category model, CCSR is the Clinical Classifications Software Refined model, and OLS is ordinary least squares.

© 2022 Ellis RP et al. *JAMA Health Forum.*

eTable 4. Mean Predicted Values and Predictive Ratios by Percentiles of Total Spending for 5 Models

		**OLS**				**Stepwise**
Total healthcare spending (not top-coded)		**Age-sex only**	**HHS-HCC**	**CCSR**	**DXI**	**DXI**
Percentile Range	Mean					
		**Mean Predicted Values**				
0-20	$9	$4,798	$2,001	$28	$328	$326
20-40	$325	$4,707	$2,314	$967	$938	$938
40-60	$1,048	$5,582	$3,349	$2,530	$2,225	$2,226
60-80	$2,958	$6,457	$5,185	$5,202	$4,691	$4,692
80-95	$10,573	$7,212	$10,177	$12,186	$12,019	$12,020
95-99	$41,086	$7,611	$24,209	$30,100	$31,933	$31,930
99-100	$182,639	$8,076	$70,832	$81,031	$89,035	$89,031
		**Predictive Ratios**				
0-20	$9	562.85	234.70	3.27	38.49	38.27
20-40	$325	14.47	7.11	2.97	2.88	2.88
40-60	$1,048	5.33	3.20	2.41	2.12	2.12
60-80	$2,958	2.18	1.75	1.76	1.59	1.59
80-95	$10,573	0.68	0.96	1.15	1.14	1.14
95-99	$41,086	0.19	0.59	0.73	0.78	0.78
99-100	$182,639	0.04	0.39	0.44	0.49	0.49

Notes: The predictive ratios are the ratios of mean predicted total healthcare spending to mean actual total healthcare spending not top-coded. The predicted values were generated from the set of models with the outcome variable total healthcare spending top-coded at $250,000. All models included age and sex as adjusters. The stepwise regression used in the final column used P<.0001. Models were estimated using the development sample with N=59,297,201. These validation sample measures used N =6,604,259. All measures are concurrent, annualized and weighted by the fraction of the year eligible. DXI is the Diagnostic Items model, HCC is the Hierarchical Condition Category model, CCSR is the Clinical Classification Software Refined model, and OLS is ordinary least squares.

© 2022 Ellis RP et al. *JAMA Health Forum.*

eMethods. DXI design features

This appendix provides additional information on specific topics related to creating and evaluating DXIs that are mentioned, but not discussed extensively, in the main text.

Non-billable diagnoses

For the existing Marketplace risk adjustment model, as well as for most performance assessment and other severity adjustments done using ICD-10-CM diagnoses, the norm is to filter out non-billable diagnoses before calculating payments.[1] For many research projects this is not always done, since even non-billable diagnoses may contain information that may be useful for prediction and/or disease surveillance. Most root codes of billable diagnoses are not billable and thus may be called "invalid diagnoses" even if more detailed codes are valid. Despite this standard policy for payment purposes, non-billable diagnoses were relatively common in the IBM Marketscan commercial claims dataset used here, comprising 1.31 percent of all diagnoses appearing on claims. This was true even after processing the claims using CMS algorithms to remove diagnoses not attached to clinician types that the Medicare program considers as valid for assigning diagnoses. We mapped all 22,512 non-billable diagnoses and 71,934 billable codes as of October 2019 (N = 94,446 in total). Subsequent to our original physician reviews of all chapters, we further included the 2020 emergency use ICD-10-CM codes for COVID-19 and vaping-related disorders.

Diagnostic information used

The DXI modifier categories and their labels were created by physicians primarily using the long and short labels of individual diagnoses and their root codes as reported on the AHRQ web site as of May 2020.[2] This project was also informed by the March 2019 release of the

© 2022 Ellis RP et al. *JAMA Health Forum.*

WHO ICD-11 coding system, scheduled for use in adopting countries in January 2022, which included a chapter for "extension codes" that can be added to ICD-11 diagnoses to capture additional clinical detail.[3] Although the content of many of these extensions was already adopted in the existing ICD-10-CM labels used for this project, in a few cases the ICD-11 naming system was relied on to standardize terminology. ICD-11 extensions were prominent in the neoplasm and injuries chapters, which used a matrix rather than a list format for presenting diagnostic information about disorders. Physician assignment of DXI modifiers was supplemented by text searches for ICD-11 extension code description strings in the US long descriptions of ICD-10-CM codes, including key words such as "bilateral", "left", "right", and "unspecified side".

Minimum sample size used for DXIs

Although it was our goal to create DXIs with at least 500 cases in them, for some sets of diagnoses grouped into DXIs this was not possible, and smaller sizes were allowed. In our development sample, only six DXI_1s had fewer than 200 cases; we excluded these six indicator variables from regressions due to concerns of imprecision. These zero or low frequency DXI_1s included Ebola, COVID-19, severe acute respiratory syndrome (SARS-2) and vaping-related disorders. These DXIs were created for their future use for disease tracking and research purposes, but no regression coefficients were assigned to any of them in our predictive models. We excluded 75 variables that were collinear with other variables in the model, including CCSR that coincided with our DXI_1s or their sums after filtering on ICD-10-CM billable codes.

Only 18.4% of all ICD-10-CM diagnoses were ultimately assigned to one DXI. eFigure 1 shows the distribution of diagnoses according to the number of DXIs (of all three types) assigned, which ranged from one to seven. eFigure 2 illustrates the similar structure of the

© 2022 Ellis RP et al. *JAMA Health Forum.*

CCSR, where multiple CCSR were allowed, but only 8.2% of diagnoses were assigned to multiple categories (ranging from one to five CCSR per diagnosis).

DXI Modifiers

In this paper we utilized only the DXI_1 main effects, reserving for future research incorporating the information in the DXI_2 modifiers or the DXI_3 scaled variables. All scaled variables were also stored as binary flags for each value as DXI_2 modifiers, which we did not attempt to aggregate to maintain at least 500 cases in each variable. Since some of the modifier information, such as initial, subsequent, and sequela, has been used in the HCC and CCSR classification systems, our DXI+CCSR system partially benefitted from such modifiers.

Negative Coefficients

In this study we estimated predictive models without imposing any restrictions on coefficients or imposing hierarchies on variables. Non-negativity restrictions are common in payment models, where researchers have often constructed models to ensure that all included coefficients are positive. Previous work has documented that the original CMS-HCC models included manual corrections to coefficients to avoid negative predictions, such as by resetting to zero one or more negative age-sex interaction terms, and constraining selected HCCs for severe developmental disabilities to be nonnegative.[4,5]

In our framework, a single negative coefficient does not necessarily imply that payment predictions will be negative. For example, if there are DXIs A, B, and C such that the variables DXI_A= DXI_B + DXI_C and the coefficient on DXI_B is less than on DXI_C, then when only DXI_A and DXI_B are included in the model then the coefficient on DXI_B will be negative,

© 2022 Ellis RP et al. *JAMA Health Forum.*

and the sum of the coefficients on A and B is positive. This will remain true when variables are simply correlated rather than perfectly colinear. This holds true in particular in our framework because we intentionally included overlapping CCSR and DXI variables: it was common that sets of detailed DXIs were a strict subset, or approximately so, of many CCSR categories.

The linear prediction models developed here illustrate the predictive power of each of the information sets examined but have not been optimized for use in payment models. Understanding the predictive power of the different diagnostic classification systems is informative even if further work is needed to ensure that predictions are non-negative.

Definition of rare diagnoses

Figure 2 in the text calculates average residuals by diagnostic frequencies for our top-coded total spending and not top-coded total spending models. To calculate these frequencies we counted for each billable diagnosis in the full sample how many enrollee-years had at least one claim with a given billable ICD-10-CM code, and divided this count by the number of enrollee-years in the sample. We grouped diagnoses by prevalence into logarithmic base 10 bins (< 1 per million, 1-10 per million, …, 10,000-100,000 per million). We generated a dataset with each distinct combination of enrollee-year and billable diagnosis in the validation sample, and then mapped onto it the residuals from that sample by enrollee-year. We then calculated the validation sample mean residual values by model and disease prevalence bin. Because the data sample included repeated draws of enrollees across calendar years, we calculated the standard errors of the sample means correcting for clustering at the enrollee-year level.

© 2022 Ellis RP et al. *JAMA Health Forum.*

[1] Kautter, J, Pope, GC, and Keenan, P. Affordable care act risk adjustment: overview, context, and challenges. *Medicare Medicaid Res Rev*. 2014; 4(3). doi:10.5600/mmrr.004.03.a02

[2] Clinical Classification Software Refine CCSR version v2020.3. ICD-10-CM Diagnosis Tool Fiscal Year 2020, Released May 2020 - valid for ICD 10-CM diagnosis codes through September 2020 https://www.hcup-us.ahrq.gov/toolssoftware/ccsr/v2020-3.zip

[3] Drösler SE, Weber S, & Chute CG. ICD-11 extension codes support detailed clinical abstraction and comprehensive classification. *BMC Med Inform Decis Mak.* 21, 278 (2021). doi:10.1186/s12911-021-01635-2

[4] Ash, A. S., Ellis, R. P., Pope, G. C., Ayanian, J. Z., Bates, D. W., Burstin, H., ... & Yu, W. (2000). Using diagnoses to describe populations and predict costs. *Health care financing review*, *21*(3), 7. PMCID: PMC4194673.

[5] Pope, GC, Kautter, J, Ellis, RP, Ash, AS, Ayanian, JZ, Iezzoni, LI, Ingber, MJ, Levy, JM, and Robst, J. Risk adjustment of Medicare capitation payments using the CMS-HCC model. *Health Care Financ Rev*. 2004; 25(4), 119. PMCID: PMC4194896.

© 2022 Ellis RP et al. *JAMA Health Forum.*
